# Integrated PRRSV prevention and control strategy based on the One Health concept: across the boundaries of virology, ecology and public health

**DOI:** 10.3389/fmicb.2025.1718572

**Published:** 2025-12-01

**Authors:** Hongbo Chen, Chengzhen Weng, Xinxin Huang, Xiaobing Li, Dianning Duan

**Affiliations:** 1 College of Life Sciences, Longyan University, Longyan, Fujian, China; 2 Fujian Engineering Research Center for Swine Disease Control and Prevention, Longyan, Fujian, China

**Keywords:** One Health, Porcine Reproductive and Respiratory Syndrome Virus, antimicrobial resistance, integrated control strategy, environmental monitoring

## Abstract

Porcine Reproductive and Respiratory Syndrome Virus (PRRSV) poses a major threat to global swine production, with substantial economic losses and serious animal welfare concerns. Although PRRSV is not considered a zoonotic agent, its control exemplifies the necessity of a One Health approach, incorporating virological, ecological, immunological, and agricultural dimensions. This article contends that the impact of PRRSV extends beyond porcine populations, significantly contributing to the emergence of antimicrobial resistance (AMR) via secondary bacterial infections and the consequent misuse of antibiotics. Moreover, the environmental persistence of the virus and its potential for indirect transmission raise critical ecological questions that remain unresolved. By synthesizing current evidence, this review delineates the complex interrelationships among PRRSV outbreaks, patterns of antimicrobial use, and environmental contamination. This study propose an integrated One Health framework for PRRSV surveillance and control, emphasizing the implementation of genomic tools, systematic environmental monitoring, and enhanced collaboration among public health, veterinary, and environmental sectors. Integrating these disciplines is crucial to alleviating the multidimensional challenges posed by PRRSV, thereby protecting animal welfare, supporting sustainable agriculture, and strengthening global public health.

## 1 Introduction

Porcine Reproductive and Respiratory Syndrome Virus (PRRSV) remains one of the most economically significant pathogens in global swine production. The virus primarily targets macrophages in pigs, weakening their immune systems and making infected animals highly susceptible to secondary infections (Sun et al., 2023). Clinical manifestations include reproductive disorders in sows (with abortion rates exceeding 30%) and respiratory diseases across all age groups, particularly acute respiratory symptoms in piglets that can result in mortality rates as high as 80%–100% (Cui et al., 2022; Yim-Im et al., 2023). Despite decades of intensive efforts by global scientific and industrial communities, controlling Porcine Reproductive and Respiratory Syndrome Virus (PRRSV) remains a formidable challenge. This stems from the virus’s high mutability, persistent infection capabilities, and complex pathogenic mechanisms. Current vaccines demonstrate limitations in providing protection, failing to stimulate potent neutralizing antibody responses while also raising safety concerns. Compounding these issues, PRRSV transmission extends beyond pig populations through environmental vectors like air and contaminated water, as well as human activities such as transportation and trade. Traditional PRRSV control strategies primarily focus on herd management measures such as vaccination, biosecurity protocols, and quarantine systems. While these approaches are crucial, they often overlook the ecological dimensions of viral transmission and human-related factors (Gao and Wen, 2025). The emergence of the One Health concept has led us to recognize that human health, animal health, and environmental health form an interconnected whole (Desvars-Larrive et al., 2024). This holistic perspective offers a fresh approach to PRRSV control, requiring interdisciplinary and cross-sectoral collaboration to address this complex challenge. This study aims to explore the application of One Health framework in PRRSV prevention and control, analyze the current gaps in monitoring and control, and propose an integrated framework integrating virology, ecology, epidemiology and public policy to provide a scientific basis for more effective and sustainable PRRSV management strategies.

## 2 PRRSV in a One Health context: beyond the pig

### 2.1 Viral characteristics and multidirectional transmission mechanisms

Porcine Reproductive and Respiratory Syndrome Virus is a membrane-coated single-stranded positive-sense RNA virus with high genetic diversity, classified into two genotypes: European (Type 1) and American (Type 2). While primarily transmitted through direct contact, studies reveal its transmission pathways extend far beyond this. The virus can spread over short to medium distances via aerosols, a transmission route confirmed by molecular evidence in field studies (Hu et al., 2023; Zhang et al., 2024). A recent study systematically summarized the stability of PRRSV under various environmental conditions, particularly highlighting temperature as a critical factor influencing its survival outside a host (Mesa et al., 2024). More importantly, PRRSV demonstrates strong environmental persistence, with studies indicating survival in feces for up to several weeks and in wastewater for several days, potentially spreading indirectly through contaminated objects, water sources, and human activities (Arruda et al., 2019; Fan et al., 2024). Studies indicate that PRRSV-infected pigs not only shed the virus through respiratory secretions but also via fecal matter, with fecal shedding posing a significant risk for environmental contamination and between-farm transmission through sewage systems, feed supplies, and transport vehicles (Mesa et al., 2024). Serological testing shows positive results for PRRSV in infected piglets from day 3 onward, with fecal-positive detection emerging by day 5. By day 7, fecal viral shedding reaches peak levels (10^3.9^ copies/0.1 g) (Arruda et al., 2019). This multi-channel shedding mechanism heightens environmental contamination risks, potentially allowing virus transmission between farms through sewage systems, feed supplies, and transport vehicles. PRRSV exhibits high mutation rates (4.7–9.8 × 10^2^/site/year) and frequent recombination events, leading to diverse lineages and sublineages. In China, lineages 1, 3, 5, and 8 co-circulate, with lineage 1 (NADC30-like) currently dominant. Recombination between sublineages (e.g., 1.8 and 8.7) further complicates control efforts (Zhang et al., 2024; Zhou et al., 2024).

### 2.2 The between PRRSV infection and antibiotic

**FIGURE 1:**
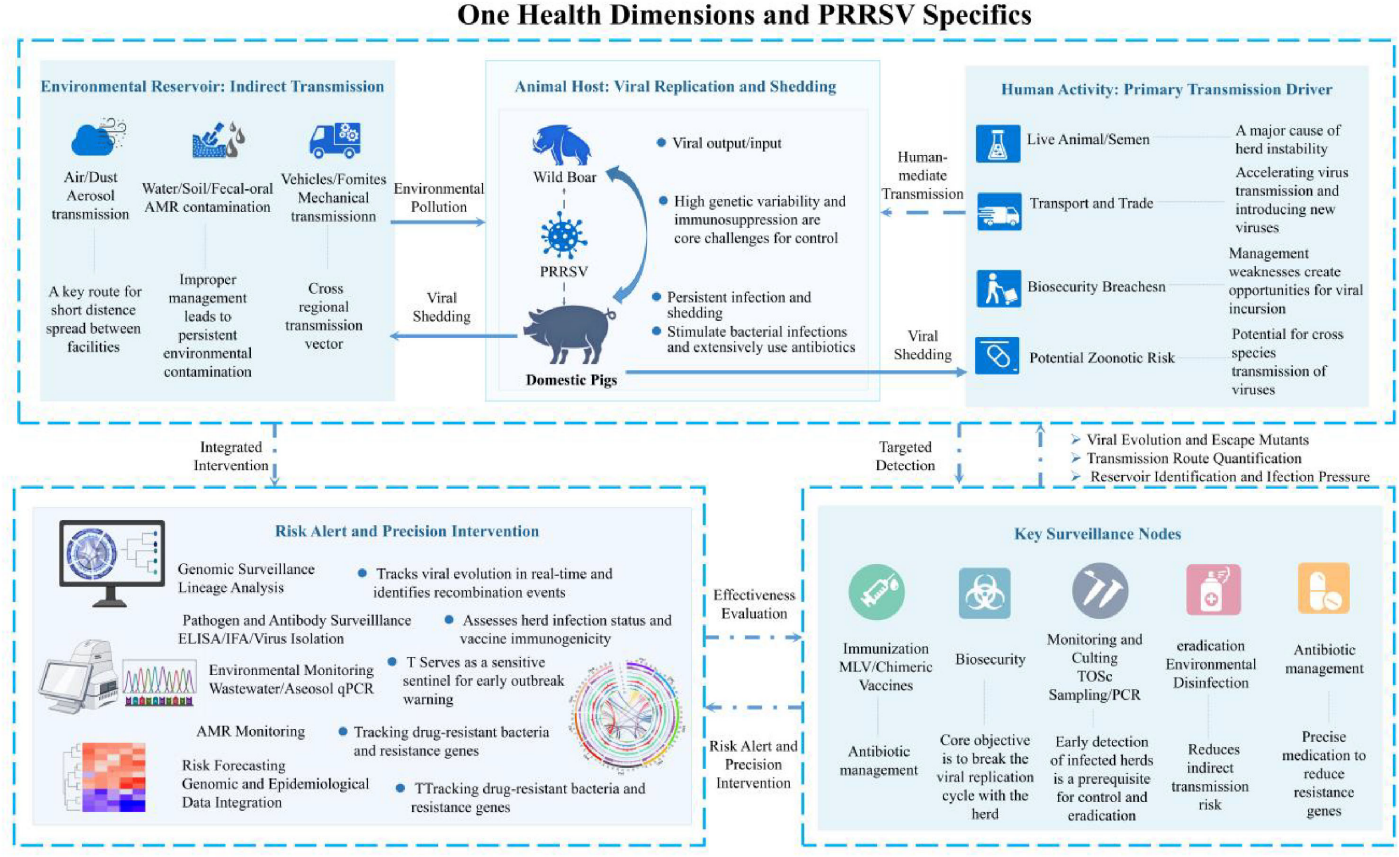
The transmission path, monitoring nodes, and intervention strategies of Porcine Reproductive and Respiratory Syndrome Virus (PRRSV) in the One Health framework.

Porcine Reproductive and Respiratory Syndrome Virus-induced immunosuppression often leads to bacterial secondary infections such as Streptococcus suis infection, Haemophilus parasuis disease, and Actinobacillus pleuropneumoniae pneumonia. Clinically, this has resulted in increased antibiotic reliance within the pig farming industry, with usage potentially rising by 30%–50% during the nursery phase. This practice not only raises production costs but also accelerates the development of antimicrobial resistance (AMR), becoming a critical concern in the One Health framework (Trevisi et al., 2022; Machado et al., 2024). It is worth noting that the abuse of antibiotics may further disrupt the intestinal microbial balance of pigs, affect immune function, and form a vicious circle. Therefore, effective control of PRRSV infection itself is one of the important strategies to reduce the use of antibiotics in pig industry, which is of great significance for alleviating the global AMR crisis (Figure 1).

### 2.3 Potential zoonotic risks and cross species transmission possibilities

Although there is currently no documented evidence of direct human infection by PRRSV, its structural similarities to human receptors and high mutation rate suggest a theoretical potential for zoonotic transmission (Gorp et al., 2010; Liu et al., 2025). Research has found that PRRSV can enter cells using receptors in pigs (such as CD163), while human cells also have similar receptors, which theoretically may have a molecular basis for cross species transmission (Gorp et al., 2010; Liu et al., 2025). On the other hand, pigs play a crucial role as “mixers” for influenza viruses in virus reassortment. The immune suppression caused by PRRSV infection may accelerate the evolution and transmission of other viruses (such as influenza virus), indirectly increasing the risk of zoonotic diseases, a concern also noted in other reviews (Lagumdzic et al., 2023). Moreover, PRRSV’s immunosuppressive effects in pigs could accelerate the evolution of co-circulating viruses, such as influenza, indirectly increasing zoonotic risks (Rajeev et al., 2020). The inherent high mutation rate and recombination capability of RNA viruses like PRRSV provide a theoretical basis for host adaptability, which is a key factor in assessing potential cross-species transmission risks from a One Health perspective (Russell et al., 2017; Ajuwon et al., 2022). In addition, PRRSV induced immunosuppression in pigs can trigger a surge of secondary bacterial infections, many of which are zoonotic, thereby posing a potential zoonotic risk from a One Health perspective (Huong et al., 2016). This indirect impact is often overlooked by traditional prevention and control strategies, but it deserves high attention under the One Health framework.

## 3 Current gaps in PRRSV surveillance and control

### 3.1 Systematic lack of environmental monitoring

The current PRRSV monitoring system mainly focuses on clinical case reports and pig herd testing, and almost completely ignores virus monitoring in the environment. Research has shown that PRRSV can survive for a long time in feces, sewage, and soil (Table 1), but the role of these environmental reservoirs in virus transmission has not been fully evaluated and monitored (Alvarez-Norambuena et al., 2025). The lack of systematic environmental sampling schemes and standardized detection methods hinders our comprehensive understanding of the transmission dynamics and environmental residual risks of PRRSV. Especially in high-density aquaculture areas, the viral load in the environment may continue to be high, leading to the risk of reinfection even on farms that implement strict biosecurity measures (Makau et al., 2021). The neglect of this environmental transmission pathway is one of the important limitations of current PRRSV control.

**TABLE 1:** Porcine Reproductive and Respiratory Syndrome Virus (PRRSV) survival time in different environmental matrices.

Environmental matrix	Survival time	Transmission risk
Airborne aerosols	Short time (minutes to hours)	Medium (short-distance transmission)
Wastewater	Up to several days	High (waterborne transmission)
Soil	Dependent on humidity and temperature	Medium
Equipment surfaces	Several hours to several days	High (human activity mediated)
Feces	Up to several weeks	High (environmental persistence)

Data are synthesized from experimental studies and reviews, including (Mesa et al., 2024) and China Porcine Reproductive and Respiratory Syndrome (PRRS) Control Network, 2021.

### 3.2 Shortcomings and limitations of genome monitoring applications

Although next-generation sequencing technology (NGS) has been widely used for monitoring many infectious diseases, it has not been fully utilized in PRRSV control. At present, whole genome sequencing (WGS) is mainly used in research scenarios and local epidemic investigations, lacking a systematic and large-scale genome monitoring network (Kim et al., 2022; Xing et al., 2022). This limitation prevents us from fully understanding the evolutionary dynamics and transmission pathways of PRRSV. Especially for the insufficient monitoring of recombinant strains, it is difficult to cope with the challenge of increasing virus diversity. The diversity of PRRSV strains has increased the difficulty of PRRS control in China, and the recombination between different strains is very serious (Xing et al., 2022). Establishing a national or even global PRRSV genome database and sharing platform is crucial for real-time tracking of virus mutations and transmission.

### 3.3 Lack of cross departmental collaboration mechanism

Porcine Reproductive and Respiratory Syndrome Virus control has traditionally been seen as the responsibility of aquaculture and veterinary departments, lacking effective collaboration with public health, environmental protection, and wildlife management departments. This departmental gap leads to a lack of overall prevention and control strategies, making it difficult to cope with complex environmental transmission and transmission risks mediated by human activities (Osemeke et al., 2025). For example, farm wastewater treatment and discharge may involve environmental protection departments, while wildlife (such as wild boars) as potential vectors of transmission may involve wildlife management departments. Lack of collaborative participation from these departments often results in blind spots and loopholes in prevention and control measures. The One Health approach emphasizes the importance of cross departmental collaboration, but in practice, this collaboration is still limited.

## 4 A proposed One Health framework for PRRSV

### 4.1 Novel integration of genomic and environmental monitoring

Establishing a comprehensive PRRSV genome monitoring network is a core component of the One Health framework. This network should integrate: clinical isolates WGS: whole genome sequencing of PRRSV in clinical samples to monitor virus variation and evolutionary dynamics; Environmental sample testing: Regularly collect environmental samples (sewage, soil, air, etc.) for virus testing and sequencing; Data sharing platform: Establish standardized data formats and sharing mechanisms to promote data exchange between different regions and institutions (Xie et al., 2021). In recent years, the development and cost reduction of metagenomics technology have provided feasible tools for environmental monitoring. Research has shown that monitoring based on metagenomics can identify epidemic signals in advance, providing valuable time windows for intervention measures. Meanwhile, integrating genomic data with epidemiological data can better track transmission pathways and identify transmission hotspots (Barton and Colijn, 2023). Our proposed framework introduces a novel integration of genomic surveillance with systematic environmental monitoring, addressing a critical gap in current PRRSV control strategies. Unlike previous approaches, our framework emphasizes real-time data sharing and metagenomic analysis to enhance early detection capabilities. The novelty of our proposal lies not in these individual technologies, but in their systematic integration across the One Health domains and the creation of a feedback loop to inform interventions, as visualized in Figure 1.

### 4.2 A novel three-tiered collaborative governance model

An effective One Health response requires the establishment of institutionalized collaboration mechanisms, that connect traditionally independent departments and disciplines. We suggest establishing a three-level collaboration framework.

Level 1: Local collaborative network, connecting farms, local veterinarians, environmental protection departments, and public health institutions, responsible for daily monitoring and information sharing.
Level 2: Regional Expert Committee, composed of virologists, epidemiologists, veterinarians, ecologists, and public health experts, responsible for data analysis and policy recommendations.
Level 3: National and international coordination agencies responsible for overall coordination, standard setting, and resource allocation.

This hierarchical structure is a key conceptual contribution of this study, designed to translate the principle of One Health into a practical, actionable governance chain for PRRSV control.

### 4.3 Differentiating existing strategies from our Integrated, risk-based approach

In situations where resources are limited, adopting risk-based precision prevention and control strategies can improve efficiency. Elements like biosecurity, vaccination, and monitoring are standard practice. However, the core of our proposed framework is the dynamic, data-driven integration of these elements based on a continuous risk assessment informed by the surveillance system (Figure 1). We propose a stratified approach where:

High risk areas (such as high breeding density and high prevalence): Implement strengthened monitoring (including environmental monitoring), strict biosafety measures, and regional vaccination plans.

Medium risk areas: Implement routine monitoring and standard prevention and control measures, with a focus on input risks.

Low risk areas: focusing on monitoring, emphasizing early detection and rapid response capacity building.

It is worth noting that the behavior of farmers is a key factor affecting the effectiveness of prevention and control. Research shows that the willingness to take action of “non-inheritor type” breeders is significantly lower than that of other types of breeders (Haile et al., 2025). Adopting differentiated communication and education strategies for farmers with different characteristics can improve the compliance and effectiveness of prevention and control measures. This represents a shift from a one-size-fits-all application of measures to a precision-guided deployment, which is a central tenet of our proposed framework.

## 5 Challenges and future directions

### 5.1 Overcoming technical challenges: the authors’ proposed solutions

The implementation of the One Health framework faces multiple challenges. Technical challenges include low virus concentrations in environmental samples, high detection sensitivity requirements, and the complexity of large-scale genomic data analysis. This requires the development of more sensitive and economical detection methods, as well as standardized bioinformatics processes (Kumblathan et al., 2021; Chen et al., 2024). We propose the development of standardized bioinformatics pipelines to manage large-scale genomic data, a solution not previously addressed in PRRSV literature.

### 5.2 Future research priorities stemming from the proposed framework

The challenges of resources and policies cannot be ignored, especially in low - and middle-income countries. Establishing a cross departmental collaboration mechanism requires policy support and financial investment, as well as overcoming cultural differences and communication barriers between departments. On a global scale, it is necessary to strengthen international cooperation and establish a PRRSV global monitoring network and data sharing platform, similar to the human infectious disease monitoring system.

Future research directions should include several critical areas that are essential for operationalizing and validating our proposed framework. Validation of the Integrated Surveillance System: Research is needed to assess the cost-effectiveness and practical implementation of the combined genomic and environmental surveillance network we propose. Operational Research on the Governance Model: The effectiveness of the three-tiered collaborative model requires empirical testing and refinement in different regional contexts. Refining the Risk Assessment Model: Future work should focus on developing and validating quantitative risk models that integrate the multi-source data (genomic, environmental, AMR) outlined in our framework to automate and improve the precision of the risk-based interventions. Virus ecology research: exploring the survival ability and transmission efficiency of PRRSV under different environmental conditions. Cross species transmission risk assessment: Evaluate the potential risks of PRRSV cross species transmission through experimental research and molecular simulations. Effectiveness evaluation of intervention measures: Compare the cost-effectiveness of different prevention and control strategies to provide scientific basis for policy formulation. Of particular concern is that climate change may affect the transmission dynamics and geographic distribution of PRRSV. Temperature, humidity, and extreme weather events may alter the survival time and transmission patterns of viruses in the environment, while also potentially affecting the distribution and abundance of vector organisms. Incorporating climate change factors into PRRSV risk models is an important direction for future research.

## 6 Final considerations

The control of Porcine Reproductive and Respiratory Syndrome Virus (PRRSV) necessitates a paradigm shift from the traditional single-pathogen-single-host model toward a holistic One Health approach. This transition requires not only technological innovation but also a fundamental evolution in mindset and collaborative mechanisms. By integrating perspectives from human, animal, and environmental health, we can achieve a more comprehensive understanding of PRRSV transmission dynamics and develop more effective intervention strategies. The One Health framework proposed in this study—the integrative feedback between surveillance and intervention, operationalized through a structured three-tiered governance model—provides a novel pathway for PRRSV management. While its implementation may face practical challenges, the potential benefits are substantial, including reduced economic impact on the swine industry, decreased antibiotic usage, mitigated antimicrobial resistance (AMR), and enhanced sustainability in agriculture and public health.

Given the evolving landscape of global swine production and the dynamic interfaces between humans, animals, and the environment, adopting a One Health approach to address animal disease threats has become increasingly imperative. PRRSV control can serve as a model for tackling complex health challenges through interdisciplinary collaboration, offering valuable insights and reference experience for the prevention and control of other emerging and endemic animal diseases.
